# Mannitol Enhances Antibiotic Sensitivity of Persister Bacteria in *Pseudomonas aeruginosa* Biofilms

**DOI:** 10.1371/journal.pone.0084220

**Published:** 2013-12-13

**Authors:** Nicolas Barraud, Alberto Buson, Wolfgang Jarolimek, Scott A. Rice

**Affiliations:** 1 Centre for Marine Bio-Innovation and School of Biotechnology and Biomolecular Sciences, The University of New South Wales, Sydney, New South Wales, Australia; 2 Pharmaxis Ltd, Frenchs Forest, New South Wales, Australia; 3 Singapore Centre on Environmental Life Sciences Engineering, and the School of Biological Sciences, Nanyang Technological University, Singapore, Singapore; Institut Pasteur, France

## Abstract

The failure of antibiotic therapies to clear *Pseudomonas aeruginosa* lung infection, the key mortality factor for cystic fibrosis (CF) patients, is partly attributed to the high tolerance of *P. aeruginosa* biofilms. Mannitol has previously been found to restore aminoglycoside sensitivity in *Escherichia coli* by generating a proton-motive force (PMF), suggesting a potential new strategy to improve antibiotic therapy and reduce disease progression in CF. Here, we used the commonly prescribed aminoglycoside tobramycin to select for *P. aeruginosa* persister cells during biofilm growth. Incubation with mannitol (10–40 mM) increased tobramycin sensitivity of persister cells up to 1,000-fold. Addition of mannitol to pre-grown biofilms was able to revert the persister phenotype and improve the efficacy of tobramycin. This effect was blocked by the addition of a PMF inhibitor or in a *P. aeruginosa* mutant strain unable to metabolise mannitol. Addition of glucose and NaCl at high osmolarity also improved the efficacy of tobramycin although to a lesser extent compared to mannitol. Therefore, the primary effect of mannitol in reverting biofilm associated persister cells appears to be an active, physiological response, associated with a minor contribution of osmotic stress. Mannitol was tested against clinically relevant strains, showing that biofilms containing a subpopulation of persister cells are better killed in the presence of mannitol, but a clinical strain with a high resistance to tobramycin was not affected by mannitol. Overall, these results suggest that in addition to improvements in lung function by facilitating mucus clearance in CF, mannitol also affects antibiotic sensitivity in biofilms and does so through an active, physiological response.

## Introduction

The formation of bacterial biofilms on living tissues often results in chronic and recurrent infections and represents a major burden for patients, sometimes leading to fatal outcomes. In cystic fibrosis (CF), colonization of the lungs by pathogenic bacteria such as *Pseudomonas aeruginosa* is the leading cause of morbidity and mortality. Immune defences and antibiotics are largely ineffective against biofilm cells because of the intrinsic high level of resistance to antimicrobial treatments characteristic of biofilms [1]. The mechanisms of this resistance remain not fully understood but appear to involve multiple factors including the maintenance of a subpopulation of persister cells, which are antibiotic-tolerant, within the biofilm [2].

Persister bacteria, which are characterized by a dormant-like state with reduced metabolic activity [3], are phenotypically distinct but genetically identical to the rest of the bacterial population. The transient nature of this physiological switch allows cells that survive antibiotic treatment to resume growth after the treatment stops, and produce a bacterial population identical to the original population consisting of both susceptible and tolerant cells [2,4]. An important trigger for the switch to a persister state appears to be the availability of nutrients and potential for metabolic activity. First, under laboratory conditions, the generation of persisters often occurs at particular growth stages that correlate with nutrient limitation [5]. Second, transcriptomic studies of persister-enriched bacterial populations revealed that genetic changes associated with the persister physiology show similarities to those induced in response to stasis and starvation [6]. Third, overall decreases in metabolic activity through nutrient starvation as well as inhibition of respiration have been shown to enhance bacterial tolerance of immune defences [7] and antibiotic treatments [8-11]. In addition to nutrient availability, the formation of persisters has also been linked to bacterial ageing and senescence [12]. The complete cellular mechanisms underlying the persister switch remain to be fully resolved, but studies so far suggest that this phenotypic switch involves several signaling systems. For instance, in the model organism *Escherichia coli* quorum sensing or general stress responses, in particular those mediated by toxin-antitoxin modules such as HipBA [13,14], as well as global regulators were linked to the regulation of the persister phenotype. Antibiotic tolerance in dormant, persister cells appears to be mediated by both passive mechanisms associated with reduced metabolic activity, such as absence of antibiotic targets linked to DNA, protein or cell-wall synthesis, or reduced molecular uptake through transporters [2], as well as active mechanisms including the production of oxidative stress defences [8].

To improve treatment of biofilm-associated infections, one potential strategy is to use combination drug therapies with multiple antibiotics that affect antagonistic phenotypes in biofilms. For instance, the antimicrobial peptide colistin has been shown to be specifically potent against bacteria with low metabolic activity. Colistin works by displacing the outer membrane lipopolysaccharides (LPS) and solubilizing the cytoplasmic membrane. Recent studies have shown that in cells with high metabolic activity, exposure to colistin triggers a modification of the LPS structure that renders the cells tolerant to the antimicrobial peptide [15]. This effect was not observed in dormant cells or cells with a low metabolism. These results prompted the investigation of novel therapies based on the use of colistin administered in combination with antibiotics effective against metabolically active cells, such as tetracycline or ciprofloxacin [15,16]. One drawback of such treatments are the potential toxicity and side effects, as is the case with colistin that is known to induce kidney damage [17]. The commonly used aminoglycoside tobramycin has also been tested in combinatorial treatments against *P. aeruginosa* infections, notably in combination with approved iron chelators [18], the β-lactam aztreonam [19] and the macrolide clarithromycin [20]. 

Given the limitations of combination drug therapies described above, it is of particular interest to develop novel approaches that prevent or revert the antibiotic-tolerant, low metabolism phenotype of persister cells, which would make them sensitive to antibiotic treatment. Recent research found that stimulating metabolic activity of *E. coli* persister cells by the addition of carbon sources such as mannitol, glucose, fructose and pyruvate could restore their susceptibility to antibiotics [21]. In these studies, addition of fructose to *Staphylococcus aureus* was also able to revert the persister phenotype. Mannitol is particularly attractive for this application, as it is a naturally occurring sugar alcohol that is already approved for use as an osmotic diuretic, and mannitol has been previously demonstrated to improve lung function by facilitating mucus clearance in patients with bronchiectasis or CF [22-26]. Its potential impact on persister cell sensitivity suggests that it may also have beneficial effects on the clearance of bacterial infections when used in conjunction with antibiotic treatment.

In this study we tested the effect of mannitol against persister cells that formed in biofilms of the main CF pathogen *P. aeruginosa* as well as two clinically relevant strains. The tolerance of persisters was investigated using the aminoglycoside tobramycin. Tobramycin is widely used to treat clinical bronchopulmonary infections and because it mainly targets ribosomal function its effectiveness is greatly reduced in the presence of persisters. Other modes of resistance towards this antibiotic have been reported that include production of inactivation factors such as periplasmic glucans, mutations of the ribosome binding sites or increased activity of efflux pumps to inhibit cellular uptake [27]. Overall our results show that addition of mannitol improves the efficacy of tobramycin, possibly via a combination of metabolic as well as, to a lesser extent, osmotic effects. This suggests that concurrent treatments of mannitol with tobramycin may improve clearance of lung infections.

## Materials and Methods

### Bacterial strains, culture media and chemicals

The laboratory strain *P. aeruginosa* PAO1 [28] was used to characterise the effects of mannitol on biofilm associated persister cells. Mannitol was also tested in two mucoid clinically relevant strains of *P. aeruginosa*, strain FRD1 [29] as well as strain 18A, which was isolated from the sputum of a chronically infected patient with CF in Tasmania, Australia [30]. A PAO1 mutant strain, *mtlD*::Tn*5*, containing a transposon Tn*5*-derived insertion element in the mannitol dehydrogenase *mtlD* gene (PA2342) was obtained from the University of Washington *P. aeruginosa* mutant two-allele library, strain PW4950 *mtlD*-C04::IS*lacZ*/hah [31]. Overnight cultures were routinely grown in Luria Bertani (LB) medium with 10 g/L NaCl with shaking at 37 °C. For antibiotic susceptibility assays, biofilm and planktonic cultures were grown in modified M9 minimal medium containing 48 mM Na_2_HPO_4_, 22 mM KH_2_PO_4_, 9 mM NaCl, 19 mM NH_4_Cl, pH 7.0, supplemented with 2 mM MgSO_4_, 100 µM CaCl_2_, and glucose at 5 mM or 20 mM [32]. Mannitol, tobramycin and carbonyl cyanide m-chlorophenylhydrazone (CCCP) were obtained from commercial suppliers (Sigma). Mannitol and tobramycin were dissolved in the biofilm M9 medium salts solution (without MgSO_4_, CaCl_2_ and glucose) as described below, and CCCP was dissolved in DMSO and diluted in M9 salts to 0.1% final DMSO concentration.

### Tobramycin minimum inhibitory concentration (MIC)

Bacterial cultures were grown in M9 medium containing 20 mM glucose with or without 40 mM mannitol from an inoculum of an overnight culture that was diluted to an OD_600_ of 0.005, in 3 mL aliquots with a serial 2-fold dilution of tobramycin. Tubes were incubated at 37 °C with shaking, and MIC values were determined as the minimum concentration of tobramycin that inhibited growth by more than 90% after 24 h of incubation.

### Biofilm multiwell plate antibiotic susceptibility assays

Biofilms were grown as previously described [32] with some modifications. Briefly, in all assays, overnight cultures were diluted to an OD_600_ of 0.005 in M9 medium containing 5 mM glucose, and 1 mL aliquots were inoculated into tissue-culture-treated 24-well plates (BD). The plates were incubated at 37 °C with shaking at 180 rpm for the duration of the experiment. After treatment, the biofilm viability was determined by a drop plate method [33]. Biofilms on the interior surfaces of the wells (surface area 4.5 cm^2^) were washed once with phosphate-buffered saline (PBS) before being resuspended and homogenised in PBS by incubating in a sonication bath (150 W, Unisonics) for 2 min [34]. Cells were then serially diluted, plated onto LB agar and colony-forming units (CFU) were enumerated after 24 h incubation at 37 °C. All assays included at minimum 2 replicates and were repeated in at least 2 or 3 independent experiments. For assays that comprised 2 independent experiments, statistical analysis was performed using all 4 individual biological replicate measurements. For assays that comprised 3 or more independent experiments, data analysis was performed using geometric means. Data were converted to a log_10_ scale and compared among groups via one-way ANOVA, followed by Tukey’s or Bonferroni’s post test for individual comparisons. Differences were deemed statistically significant at a P value < 0.05.

Persister bacteria in biofilms were identified as previously described [21,35] with some modifications. Biofilms were grown as described above in the absence of antibiotic for 5 h or 20 h, for *P. aeruginosa* PAO1 young or established biofilms, respectively, for 5 h for *P. aeruginosa* FRD1 or for 24 h for *P. aeruginosa* 18A biofilms to allow for the development of sufficient biomass, before being treated with tobramycin at various final concentrations for up to 3 h. Biofilm CFU were subsequently enumerated as described above. Based on the results of the identification of persister bacteria in biofilms, tobramycin concentrations of 80 mg/L for *P. aeruginosa* PAO1 and FRD1 or 400 mg/L for *P. aeruginosa* 18A were selected for combined treatments with mannitol.

The effect of mannitol on persister cells was tested both for its ability to prevent their formation and to revert the phenotype. To determine the impact on the formation of persisters, *P. aeruginosa* biofilms were grown as described above but with or without mannitol in the M9 medium from the beginning of growth. After 5 h, tobramycin was added directly to the wells and the biofilms were incubated for a further 2 h, which is sufficient to select for persisters cells based on initial experiments and appears consistent with data previously reported [21]. The biofilm viability was then assessed by quantifying CFU. To determine whether mannitol could revert the formation of persister cells, biofilms were grown in the absence of any treatment for 5 h, 20 h or 24 h, for PAO1 and FRD1 young biofilms, PAO1 established biofilms or 18A biofilms, respectively, and treated with or without tobramycin for 1 h to select for persister cells. Then, 10 µL of a stock solution at the appropriate concentration of mannitol, glucose or NaCl, with or without CCCP were added directly to the wells, and the cultures now containing both tobramycin and mannitol or controls were incubated for a final 1, 2 or 3 h, before assessing the biofilm viability.

For microscopy analysis, *P. aeruginosa* PAO1 biofilms were grown in glass-bottom 24-well plates (MatTek Corporation, Ashland MA, USA) with or without mannitol from the beginning of growth as described above. After 6 h or 24 h incubation, biofilms were rinsed twice with PBS before being stained with LIVE/DEAD *Bac*Light bacterial viability kit reagents (Molecular Probes) and visualised by using inverted confocal microscopy (Leica TCS SP5).

### Microfermenter continuous flow biofilm dispersal assays

To determine whether mannitol may cause dispersal of biofilms, *P. aeruginosa* PAO1 biofilms were grown in microfermenters with a continuous flow of fresh M9 minimal medium with 2 mM glucose at 37 °C as previously described [36]. After 24 h, glucose or mannitol were added to the biofilm medium and the release of dispersal cells was monitored by collecting the biofilm effluent every 10 min and measuring the OD_600_.

## Results

### Biofilm-associated persister bacteria in *P. aeruginosa*

**Figure 1:**
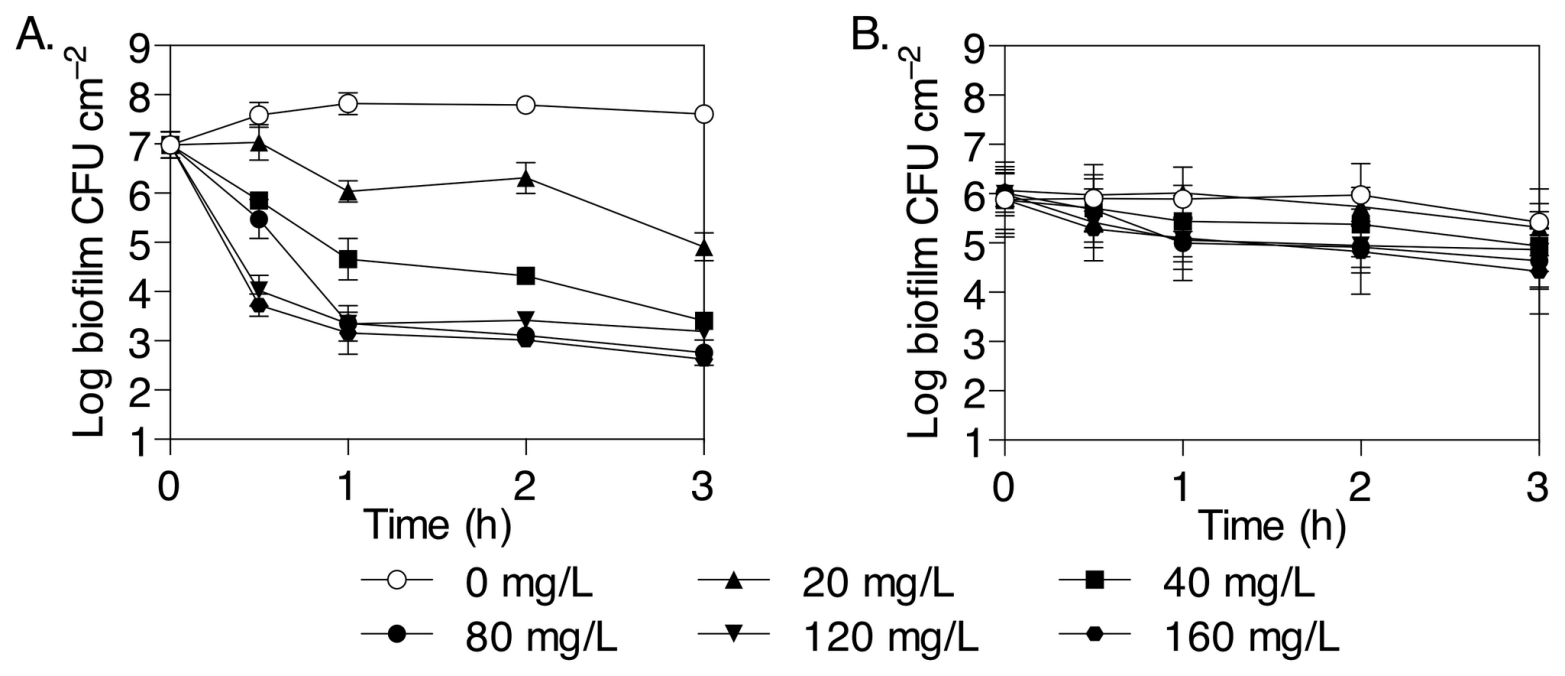
Biofilm-associated persister bacteria in *P. aeruginosa*. (A) Young *P. aeruginosa* biofilms pre-grown for 5 h in the absence of antibiotic before being treated with tobramycin at 0-160 mg/L for 1-3 h. Biofilm viability was analysed by the drop plate method and CFU counts. Error bars indicate standard error of the geometric mean (SEM, n = 3). (B) Same as in (A) but established *P. aeruginosa* biofilms were pre-grown for 20 h in the absence of antibiotic before being treated with tobramycin. Error bars indicate standard deviation (SD, n = 4).

To investigate the presence of persister cells in *P. aeruginosa* biofilms, the bactericidal activity of the aminoglycoside tobramycin was tested against biofilms grown in batch culture in minimal medium with glucose as carbon source. To assess the kinetics of tobramycin killing of biofilms, different exposure times were tested against young and established biofilms. At all concentrations, maximal reduction in CFU was observed within the first hour of exposure both in young (Figure 1A) and established biofilms (Figure 1B). The killing effect of tobramycin on young *P. aeruginosa* biofilm bacteria appeared to follow a biphasic kill curve and was dose-dependent when tobramycin was administered at concentrations between 0 and 80 mg/L, but higher concentrations did not cause any further killing. The initial dose of 40 mg/L induced a 3 log decrease in CFU and 80 mg/L (170 µM), corresponding to 64 × MIC (Table 1), increased this reduction to a 4 log decrease in CFU. No further significant reduction could be obtained after treatment with higher concentrations of tobramycin (Figure 1A), which indicates that the remaining population of 1.2 × 10^3^ CFU cm^-2^ cells after 1 h exposure to tobramycin were tolerant to the antibiotic. When established biofilms were treated with tobramycin, a biphasic kill curve was also observed but the extent of killing was much reduced compared to young biofilms. Thus, 80 mg/L tobramycin induced a 1 log decrease in CFU after 1 h, and the remaining population of 2.0 × 10^5^ CFU cm^-2^ cells was not further reduced by higher concentrations of tobramycin, or longer incubation times up to 3 h. To confirm that the tolerant bacteria were persisters and not a subset of resistant mutants, the resuspended biofilm cells were grown overnight and tested for tobramycin MIC. The results show that persister cells were not modified from the original inoculum and that the MIC for tobramycin, 1.25 mg/L, was identical to the initial sensitivity of the parent strain (Table 1). Thus, according to the established method for identifying persister cells [35], it is clear that the tobramycin tolerant population observed here consisted of persister cells.

**Table 1:** MICs of tobramycin for *P. aeruginosa* strains used in this study and persisters cells generated from the corresponding strains.

Bacterial strain	Ref	MIC**^*a*^** (mg/L)		MIC persisters**^*b*^** (mg/L)
		Without mannitol	With 40 mM mannitol	
*P. aeruginosa*				
PAO1	[28]	1.25	1.25	1.25
FRD1	[29]	2.5	2.5	2.5
18A	[30]	20	20	20

^***a***^ MIC for a culture inoculated with the parent strain.

^***b***^ MIC for a culture inoculated with biofilm persister cells that survived after tobramycin treatment.

### Mannitol prevents formation of persister cells

**Figure 2:**
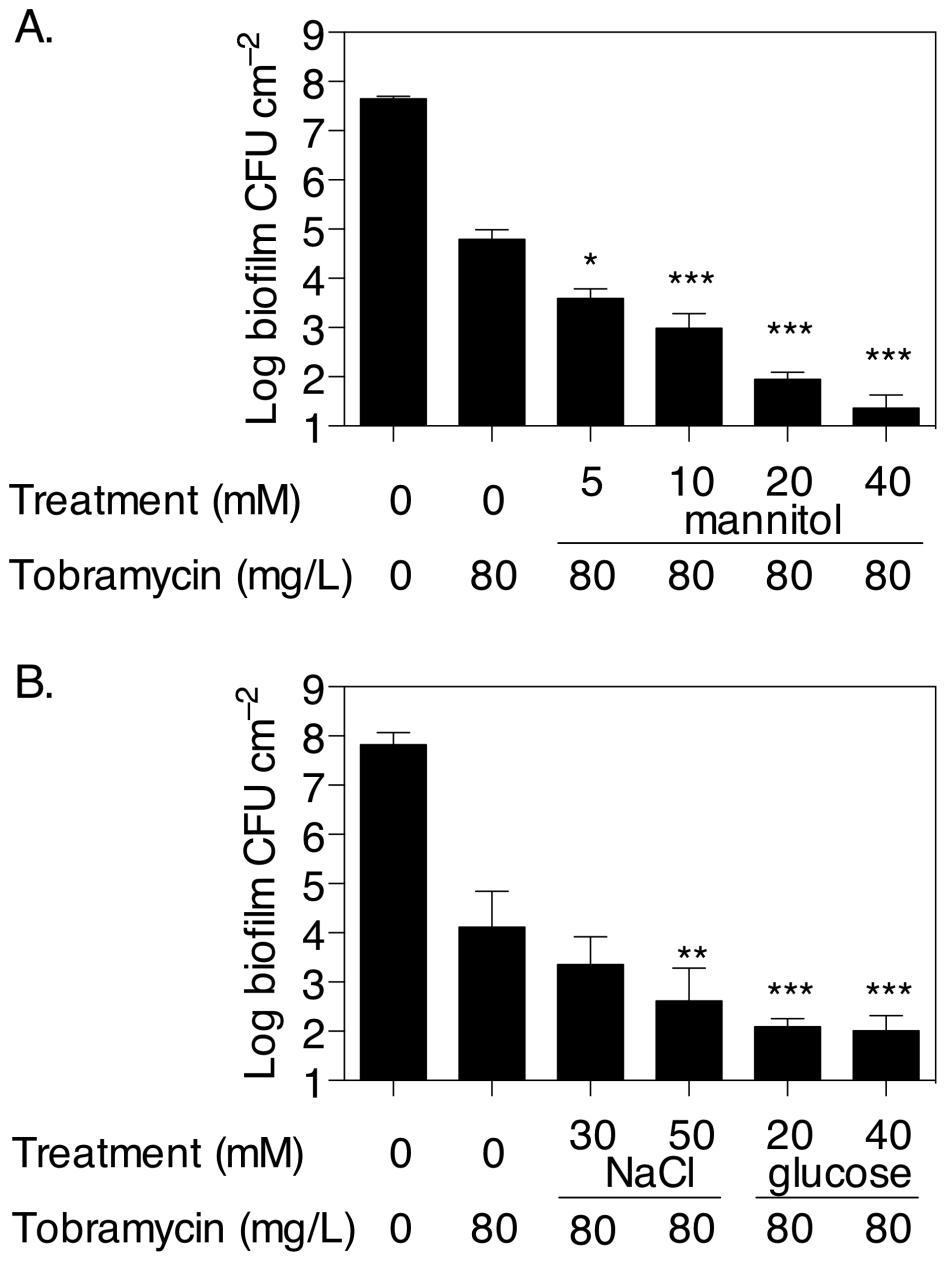
Mannitol prevents the formation of persister cells in *P. aeruginosa* biofilms. (A) Biofilms were grown in multiwell plates for 5 h in M9 minimal medium with glucose as a carbon source, with or without mannitol at 5-40 mM. Tobramycin was then added to the culture medium to 80 mg/L and the plates were incubated for a further 2 h before enumerating CFU. Error bars indicate SEM (n = 3). (B) To check for substrate or osmotic effect, mannitol was replaced with NaCl or additional glucose at various concentrations. Error bars indicate SD (n = 4). Asterisks indicate statistically significant difference of combination treatments versus tobramycin only (*, *P* < 0.05; ***, *P* < 0.001).

The effect of mannitol on *P. aeruginosa* biofilm persister cells was first assessed by growing biofilms in the presence or absence of mannitol at 0-40 mM from the beginning of the experiment and with glucose as the carbon source for 5 h, before treating with 80 mg/L tobramycin. Exposure of the biofilm to 40 mM mannitol, the highest concentration tested, significantly decreased biofilm viability (*P* < 0.001) to less than 30 CFU cm^-2^ compared to 6.2 × 10^4^ CFU cm^-2^ in the absence of mannitol. Thus, the addition of mannitol enhanced the antibiotic effect by 99.96% (Figure 2A). The data demonstrate that mannitol, which was added before and remained present during tobramycin treatment, prevented the formation of persister cells and increased the sensitivity of the biofilm to tobramycin. This effect was concentration dependent, at 5-40 mM mannitol. Addition of mannitol did not change the MIC of *P. aeruginosa* (Table 1). To control for either osmotic effects or for nutrient effects, biofilms were also exposed to NaCl or additional glucose (Figure 2B). The results indicated that at an osmolarity similar to the highest concentration of mannitol (40 mM), which contributed 40 mOsm/L to the medium, NaCl had little effect on tobramycin sensitivity in *P. aeruginosa* biofilms (data not shown), hence 30 and 50 mM NaCl were added to the medium, which are equivalent to 60 and 100 mOsm/L, respectively. At 100 mOsm/L NaCl or 40 mM glucose there was a 96.5% and 99.3% reduction in persister cells by NaCl and glucose, respectively, compared to tobramycin alone treatments. The effects of NaCl were significantly less than that observed for the mannitol at 40 mM (*P* < 0.05), but the effects of additional glucose, although less, were not significantly different from those of mannitol (*P* ≥ 0.1).

### The persister phenotype can be reverted by mannitol in both young and established biofilms

**Figure 3:**
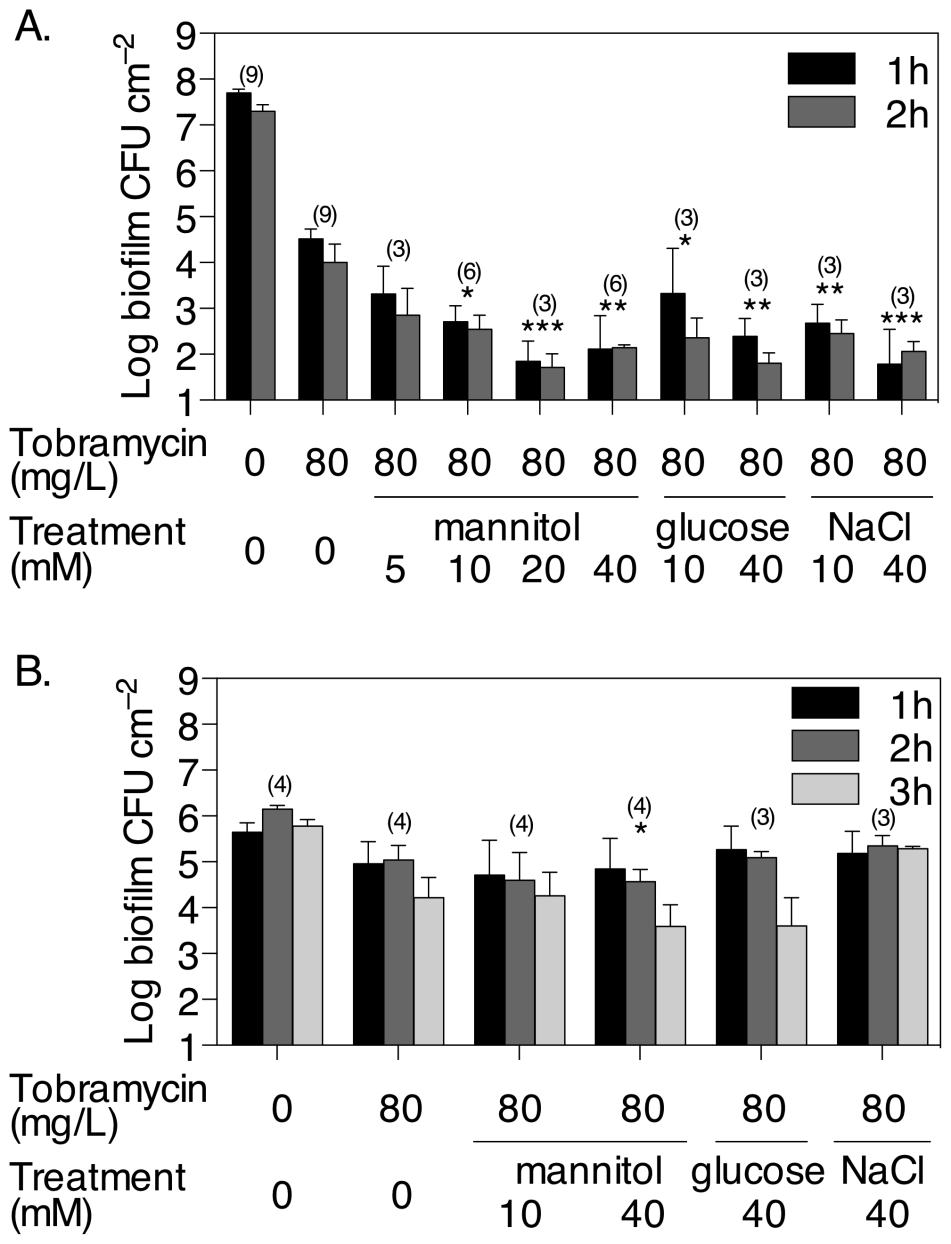
Exposure to mannitol reverses the persister phenotype in *P. aeruginosa* biofilms. (A) Young, 5 h old biofilms grown in multiwell plates were first treated with or without 80 mg/L tobramycin for 1 h to select for persister cells. Then mannitol was added to the culture and the plates were incubated for a further 1-2 h, before enumerating CFU. Glucose and NaCl were used in place of mannitol to check for substrate or osmotic effects. (B) Established, starving biofilms grown for 20 h in the absence of treatment were exposed or not to tobramycin for 1 h. Mannitol, glucose or NaCl were then added the cultures for a further 1-3 h, before analysing CFU. Error bars indicate SEM (n is indicated in parentheses for each set of samples). Asterisks indicate statistically significant difference of combination treatments versus tobramycin only (*, *P* < 0.05; **, *P* < 0.01; ***, *P* < 0.001).

The effect of mannitol on persister cells was further assessed by examining its capacity to revert persister cells in pre-grown biofilms. First, young *P. aeruginosa* biofilms were pre-grown for 5 h and treated with tobramycin for 1 h to select for persister cells, after which time mannitol was added to the culture medium. Addition of mannitol was found to significantly revert, in a concentration dependent manner, the persister phenotype of biofilm cells remaining after tobramycin treatment. At 40 mM and after 1 h, mannitol exposure resulted in less than 30 CFU cm^-2^ compared to 3.3 × 10^4^ CFU cm^-2^ in the absence of mannitol. After 2 h, the addition of mannitol increased the antibiotic effect by 99.5% (Figure 3A) when treating young biofilms. Addition of NaCl also significantly increased the efficacy of tobramycin. Again, the concentrations at which NaCl triggered an effect corresponded to much higher osmolarities compared to the effective concentrations of mannitol. When NaCl and mannitol were tested at the same osmolarity (10 mM NaCl and 20 mM mannitol), NaCl had a substantially (6-fold) lower effect compared to 20 mM mannitol. At high NaCl osmolarities, which were 2-fold higher than the osmolarity of effective mannitol concentration, the maximal inhibitions were similar compared to 40 mM mannitol, and there was no statistically significant difference for the high NaCl concentration relative to the mannitol (*P* ≥ 0.1). Addition of glucose at 40 mM resulted in a 99.4% reduction of persisters after 2 h, a similar outcome compared to mannitol and high concentrations of NaCl (*P* ≥ 0.1).

Secondly, the effect of mannitol on established biofilms was assessed. Biofilms were grown in batch culture for 20 h. Previous studies showed that at this time, glucose has been entirely consumed and biofilms have undergone dispersal events after the onset of starvation [37]. After 1 h exposure to tobramycin, 9.1 × 10^4^ CFU cm^-2^ remained in the biofilm, compared to 4.4 × 10^5^ CFU cm^-2^ in untreated biofilms, indicating that 21% of bacteria in established biofilms were persisters (Figure 3B). In contrast, only 0.1% of the population of young biofilms were persisters (Figure 3A). The addition of mannitol to established biofilms increased the efficacy of tobramycin, with 40 mM mannitol reducing the biofilm to 3.9 × 10^3^ CFU cm^-2^ after 3 h, representing a 77% increased killing efficacy compared to tobramycin alone (Figure 3B). Glucose at 40 mM also increased the efficacy of tobramycin by 76% after 3 h. Interestingly, the addition of NaCl had no effect.

### Reversion of persister cells by mannitol is not linked to biofilm dispersal and requires energy

**Figure 4:**
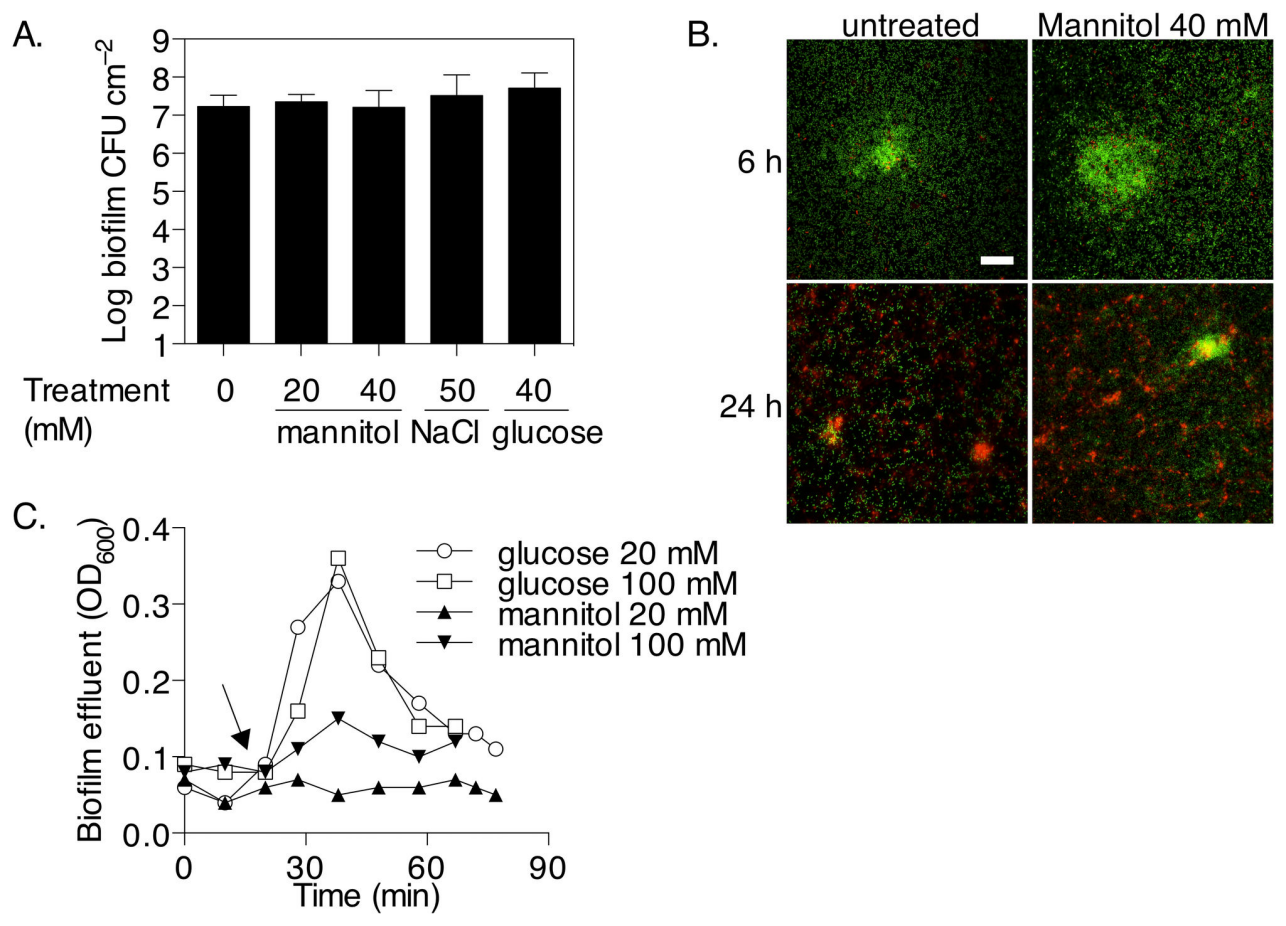
Mannitol alone does not induce biofilm dispersal or decrease in biofilm CFU counts. (A) Biofilms were pre-grown for 5 h in microtitre plates in the absence of any treatment. Then individual treatments with mannitol, glucose, NaCl or tobramycin as a positive control were added to the wells and the plates incubated for further 2 h before analysing biofilm viability by the drop plate method and CFU counts. Error bars indicate SD (n = 4). (B) Microscopic images of LIVE/DEAD stained *P. aeruginosa* biofilms grown at the bottom of 24-well plates for 6 h or 24 h in the presence or absence of 40 mM mannitol. Live cells appear green, dead cells appear red. Note that the DEAD stain is also known to bind extracellular DNA in the biofilm matrix [51]. Bar, 20 µm. (C) *P. aeruginosa* biofilms were cultivated in continuous flow microfermenters with glucose at 2 mM. After 1 day of growth, the medium inlet was supplemented (arrow) with glucose at 20 mM (opened circles), 100 mM (opened squares), or mannitol at 20 mM (filled upright triangles) or 100 mM (filled inverted triangles) and the release of dispersal cell was monitored by measuring the OD_600_ of the effluent runoff.

The sudden supplementation of nutrients to established biofilms was previously found to induce dispersal [38] and induction of biofilm dispersal has been linked to increased antibiotic susceptibility [39]. Because mannitol can be used for metabolism in *P. aeruginosa*, we tested whether its addition to biofilms may also cause dispersal. In the absence of tobramycin, neither the addition of mannitol, glucose nor NaCl resulted in decreases in biofilm CFU compared to control biofilms in the multiwell plate assays (Figure 4A). Microscopy analysis of 6 h biofilms indicated that the wells of the multiwell plates were covered with bacteria and small clusters of cells or microcolonies could be observed (Figure 4B). Addition of mannitol did not decrease the amount of cells attached on the bottom surface of the wells in both young and established biofilms (Figure 4B) which supports that mannitol is non-toxic and does not have any effect on biofilm dispersal or prevention of biofilm formation. To confirm that mannitol does not have any effect on dispersal, *P. aeruginosa* PAO1 biofilms were grown for 1 day in continuous flow microfermenters and exposed to either mannitol or glucose as a positive control at 20 mM and 100 mM. While glucose at both concentrations resulted in biofilm dispersal as previously reported [38], addition of mannitol at 40 mM had no effect on dispersal and addition of mannitol at 100 mM could induce some dispersal but to much reduced extent compared to glucose (Figure 4C).

**Figure 5:**
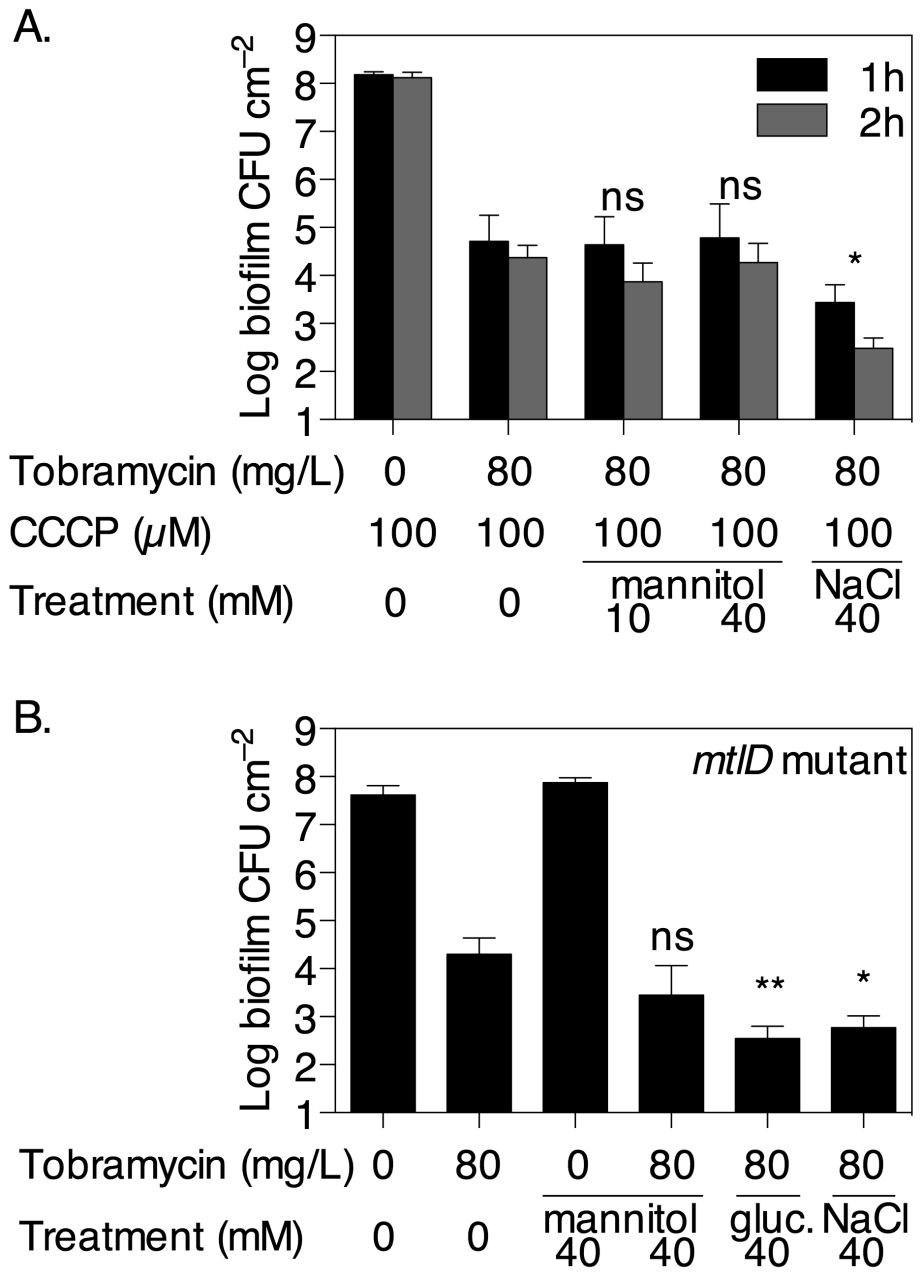
Mannitol reverts persister *P. aeruginosa* in biofilms mainly by increasing their metabolic activity. (A) Addition of the PMF inhibitor CCCP blocks the effect of mannitol on tobramycin susceptibility. Biofilms were grown in multiwell plates for 5 h as described in Figure 3A except that after treatment with tobramycin for 1 h, CCCP was added at 100 µM at the same time as mannitol to the cultures, and the plates were incubated for a further 1-2 h before enumerating CFU. Error bars indicate SEM (n = 3). (B) Mannitol has limited effect on biofilms of a mannitol dehydrogenase, *mtlD*, mutant strain. *mtlD*::Tn*5* mutant biofilms were grown as described above and treated with tobramycin for 1 h, then mannitol, glucose or NaCl were added to the wells and the plates incubated for a further 2 h before enumerating CFU. Error bars indicate SD (n = 4). ns indicates no significant difference (*P* ≥ 0.1) and asterisks indicate statistically significant difference (*, *P* < 0.05; **, *P* < 0.01) of combination treatments versus tobramycin and CCCP only in panel A, or versus tobramycin alone only in panel B.

To assess whether the reversion of persister cells by mannitol required energy, the proton-motive force inhibitor (PMF) CCCP was added at the same time as mannitol to pre-grown young biofilms that had been treated or not with tobramycin. The addition of 100 µM CCCP did not appear to increase the number of tobramycin persisters in the absence of mannitol (Figures 3A and 5A). In the presence of CCCP, the addition of mannitol, even at 40 mM which was shown above to revert persister cells, could not further increase killing compared to treatments with tobramycin and CCCP alone (Figure 5A). In contrast, addition of CCCP did not prevent NaCl to increase killing of biofilm bacteria by tobramycin. Further, the effect of mannitol was also tested on young biofilms of a PAO1 *mtlD*::Tn*5* mutant strain, unable to express the mannitol dehydrogenase and thus metabolise mannitol. The results show that 40 mM mannitol only had a limited, not statistically significant, effect on the reversion of persister cells in biofilms of this strain, improving tobramycin killing by only 86.2% compared to 98.6% in PAO1 wild type biofilms. In contrast, addition of 40 mM glucose or NaCl reverted persisters to a similar extent as the wild type strain, increasing killing by 98.2% and 97.0%, respectively (Figure 5B). Taken together, these results strongly suggest that mannitol reverts persister bacteria mainly by inducing metabolism and generating a PMF in these cells.

### Mannitol can reverse persister cell formation in a *P. aeruginosa* mucoid clinically relevant strain

**Figure 6:**
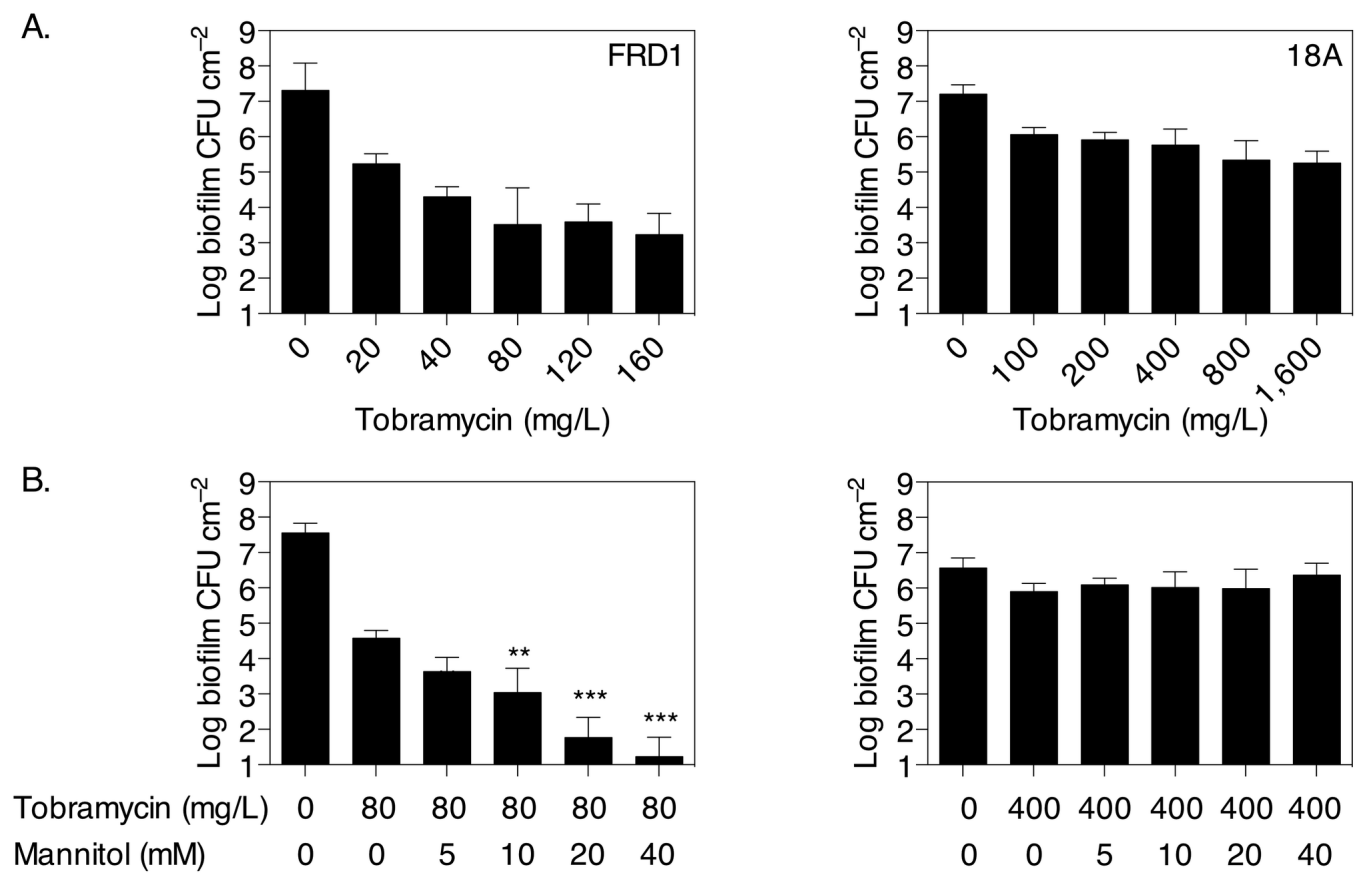
Mannitol increases the efficacy of tobramycin against clinically relevant strain *P. aeruginosa* FRD1, but not tobramycin resistant *P. aeruginosa* 18A, which was isolated from a CF patient. (A) Biofilms were grown in multiwell plates for 5 h (FRD1) or 24 h (18A) at which time tobramycin was added to the cultures at various concentrations. The plates were incubated for a further 2 h before enumerating CFU. (B) Biofilms were grown for 5 h (FRD1) or 24 h (18A) in the absence of antibiotic, before being treated with or without tobramycin at 80 mg/L (FRD1) or 400 mg/L (18A) for 1 h to select for persisters. Then mannitol was directly added to the wells at 0-40 mM, and the plates were incubated for a further 2 h before analysing CFU. Error bars indicate SD (n = 4). Asterisks indicate statistically significant difference of combination treatments versus tobramycin only (**, *P* < 0.01; ***, *P* < 0.001).

The results presented thus far strongly suggest that mannitol may be useful for the eradication of *P. aeruginosa* biofilm related infections. To further assess the efficacy of this treatment, we investigated its effect on two clinically relevant strains. The mucoid strain *P. aeruginosa* FRD1 was observed to respond similarly to tobramycin as the PAO1 strain with concentrations of tobramycin ≥80 µg/ml unable to cause further reduction in CFU indicating persister cells (Figure 6A), and the addition of mannitol could alleviate the persister phenotype (Figure 6B). When the CF isolate *P. aeruginosa* 18A was tested, exposure to tobramycin up to 1,600 mg/L, thus 80 × MIC (Table 1), showed only a 2 log reduction in CFU (Figure 6A), indicating that biofilms of this strain were highly tolerant to tobramycin relative to PAO1 and FRD1, which is consistent with a higher MIC of tobramycin in this strain compared to PAO1 and FRD1 (Table 1). The addition of mannitol to 18A biofilms had no significant effect on the antibiotic resistant population at any concentration tested (*P* ≥ 0.1, Figure 6B).

## Discussion

The presence of drug tolerant, persister bacteria in biofilms causes major health concerns leading to chronic infections, which require frequent hospital visits for intensive therapy or at times cannot be controlled. Here we found that mannitol, one of the most abundant sugar alcohols in nature, could revert the persister phenotype of *P. aeruginosa* biofilms and increase, by several orders of magnitude, the efficacy of the antibiotic tobramycin. This effect of mannitol was consistent with published data obtained with persisters of other bacterial strains [21]. Because another metabolic substrate, glucose, similarly improved the outcome of antibiotic therapy and the effects of mannitol were blocked by a PMF inhibitor, it is likely that the primary effect is metabolic. Further, NaCl at osmolarities higher than those used here for mannitol, also showed a positive outcome on the efficacy of tobramycin but to a lesser extent than observed for the mannitol treatments. Taken together, the data suggest that mannitol acts primarily through a metabolic effect and an increase in osmolarity may also contribute to its impact on the tobramycin sensitivity in biofilms.

### Persister bacteria are abundant in biofilms

Treatments with increasing concentrations of tobramycin revealed that *P. aeruginosa* biofilms harbour a high number of persister bacteria. Young, growing biofilms, which were covered with bacteria and small clusters of cells or microcolonies (Figure 4B), were found to typically contain 5 × 10^4^ CFU cm^-2^ persisters, representing 0.1% of the total biofilm CFU. Established, starving biofilms, which contained lower total CFU (Figure 3) and had less cells on the wells bottom surface compared to young biofilms (Figure 4B) due to dispersal events [37], were far more resistant to tobramycin treatment compared to young biofilms. They harboured 1 × 10^5^ CFU cm^-2^ persisters, representing up to 21% of the total population. The biofilm-associated persisters occur in much higher numbers than has been observed for mid-log phase cultures, where persisters typically represent 0.001% of the overall bacterial cells [2]. In biofilms, the presence of steep nutrient gradients can lead to nutrient depletion even in growing biofilms [40-42], which supports a link between low nutrient availability, starvation and the persister phenotype. While it remains unclear if the formation of persisters was induced upon treatment with the antibiotic in our experiments, as it was observed previously using ciprofloxacin [43], or if persisters were already present in biofilms, our data strongly suggest that mature biofilms favour the generation of persisters. The failure of higher concentrations of tobramycin to clear the persister cells from the biofilm demonstrates that increasing the concentrations of antibiotics is unlikely to have any beneficial effect for the removal of biofilms. Moreover, caution should be taken as exposure to tobramycin alone has previously been found to result in an increase in biofilm formation in several CF isolates of *P. aeruginosa* [44].

### Mannitol has a positive effect on biofilm clearance by antibiotics

Mannitol increased the efficacy of tobramycin against *P. aeruginosa* biofilms in a concentration dependent manner, and up to 3 log at 40 mM. Dry powder mannitol is used as a mucus-clearing agent in CF patients. While the concentration of mannitol is constantly changing in the airway surface liquid due to its water absorbing activities initial concentrations of mannitol are well above 100 mM. Hence the concentrations studied here appear to be well within the therapeutically relevant and achievable range. 

The osmolarity of fresh biofilm M9 medium containing 5 mM glucose is 250 mOsm/L and the addition of 40 mM mannitol increases the osmolarity of the medium by 40 mOsm/L, which is the same as for 20 mM NaCl. In this system, increases in osmolarity by 80-100 mOsm/L using higher concentrations of NaCl resulted in improved killing by tobramycin. This effect appeared to be independent of a PMF as it was not reverted by addition of CCCP (Figure 5A), which suggests that it likely resulted from increased drug uptake induced by osmotic changes. This observation is consistent with recent findings that the addition of osmolytes prevented persister formation in *E. coli* [45]. However, lower concentrations of NaCl had little effect. This suggests that osmotic stress may contribute to antibiotic mediated killing of persisters but was not the main factor underlying the effect of mannitol in these experiments. In contrast, the metabolic substrate glucose, at concentrations similar to mannitol, also had a strong impact on persisters. While glucose was already present in the growth medium, the addition of supplementary glucose likely alleviated nutrient depletion due to gradients within the biofilm and increased metabolic activity in cells previously experiencing low glucose levels. Further, the effect of mannitol appeared to be dependent on increased metabolic activity as its effects were abolished in the presence of the PMF inhibitor CCCP. It should be noted that exposure of the biofilm to tobramycin in the presence of CCCP did not change the number of persister cells identified, approximately 10^5^ CFU cm^-2^ compared to tobramycin alone treatments. The importance of a metabolic effect of mannitol on the reversion of persister cells was further supported by the observation that biofilms of a mannitol dehydrogenase mutant strain were not significantly affected by mannitol but still showed increased sensitivity to tobramycin after exposure to glucose and to a lesser extent NaCl (Figure 5B). This strongly suggests that at 40 mM (~0.7% w/v) mannitol reverts the persister phenotype in *P. aeruginosa* biofilms mainly by inducing a metabolic pathway and generating a PMF, in a similar fashion as it was demonstrated in planktonic *E. coli* bacteria [21]. The downstream effect on tobramycin toxicity may be via increased uptake of the aminoglycoside as was suggested in *E. coli* [21]. Another possibility is that exposure to mannitol in *P. aeruginosa* persisters may inhibit starvation-induced defences such as the stringent response, thus leading to decreased production of antioxidant such as catalase. In turn, reduced antioxidant defence could increase the potency of antibiotics such as tobramycin that are known to induce oxidative stress [46,47]. Mannitol is known to have antioxidant properties [48] but may not have any impact on tobramycin-mediated oxidative stress.

In our experiments, beneficial effects of mannitol on tobramycin sensitivity could be observed in biofilms of two strains tested, *P. aeruginosa* PAO1 and FRD1, however, no effect was found when mannitol was added to biofilms of the P. *aeruginosa* strain 18A which was recently isolated from a CF patient. The resistance to tobramycin of *P. aeruginosa* 18A biofilms suggests that *P. aeruginosa* 18A may also have a multi-drug resistant phenotype, consistent with an MIC for tobramycin more than 15 fold higher compared to that of PAO1. This finding supports the assumption that a multi-drug resistance phenotype may be the consequence of a genetically encoded mechanism, such as increased expression of efflux pumps or enzymatic deactivation of antibiotics, the efficacy of which are unlikely to be influenced by the presence or absence of a metabolite. In contrast, the persister phenotype is a transient state that develops over the growth of the bacterial population, does not affect all the cells and can be reverted by exposure to mannitol. Other chemical compounds have recently been found that are able to revert the persister phenotype. The synthetic brominated furanone (Z)-4-bromo-5-(bromomethylene)-3-methylfuran-2(5H)-one, a known quorum sensing inhibitor, was found to increase susceptibility of *P. aeruginosa* towards tobramycin as well as the fluoroquinolone ciprofloxacin, and the effect was suggested to involve direct activation of transport systems rather than operate via a quorum sensing pathway [49]. In another study, a screen of 6,800 compounds from a chemical library identified 3-[4-(4-methoxyphenyl)piperazin-1-yl]piperidin-4-yl biphenyl-4-carboxylate, a polycyclic small molecule, as a potent treatment effective at reverting *E. coli* persisters into fast growing cells and restoring their susceptibility to the fluoroquinolone norfloxacin [50]. Therefore several strategies may be useful in order to combat persister cells and antimicrobial resistance, and increasing metabolic activity of persister cells appears to be a key element to improve antibiotic therapy outcomes.

Overall our results, using clinically relevant strains and the standard aminoglycoside tobramycin, suggest that the use of mannitol as an adjuvant to antibiotic therapy may improve the clearance of recalcitrant biofilm infections in addition to improving lung function. In a recent phase III efficacy study of mannitol in CF patients, there was no evidence that the reduction of pulmonary exacerbation incidence observed in the mannitol group was associated with a change in bacterial load in sputum after several weeks exposure to mannitol [26]. However mannitol was given in addition to best care, which includes regular antibiotic treatment and sputum collections were not synchronised with antibiotic applications. Furthermore, the analysis of microorganisms dispersed in sputum may not accurately reflect the efficacy of treatments against biofilms still residing in the lungs. Further studies will be needed to better understand the impact of combined treatments of mannitol and antibiotics for the removal of biofilm infections *in vivo*.
